# Geriatric Models of Surgical Care: A Scoping Review

**DOI:** 10.1111/jan.70518

**Published:** 2026-02-02

**Authors:** Sarah Hughes, Ut Bui, Clint Douglas, Andrea Taylor

**Affiliations:** ^1^ Surgical and Perioperative Services Royal Brisbane and Women's Hospital Herston Queensland Australia; ^2^ School of Nursing Queensland University of Technology (QUT) Kelvin Grove Queensland Australia; ^3^ Office of Nursing and Midwifery Services Metro North Hospital and Health Service Herston Queensland Australia

**Keywords:** acute care, frailty, geriatrics, hospital, model of care, multidisciplinary care, older adult, perioperative care, surgery

## Abstract

**Aim:**

To synthesise literature on hospital‐based geriatric models of care for older adults undergoing surgery, examining structures, team composition, governance and nursing contributions.

**Design:**

Scoping review.

**Methods:**

Following JBI methodology, two reviewers independently screened articles against eligibility criteria (Population: adults ≥ 65 years, Concept: multidisciplinary geriatric surgical care model; Context: acute hospital settings), with conflicts resolved by a third reviewer. Data were extracted and charted for descriptive synthesis.

**Data Sources:**

Six databases (CINAHL, MEDLINE, Embase, Scopus, AgeLine, Cochrane Library) searched for studies published between January 2015 and February 2025.

**Results:**

Of 2753 records identified, 81 studies were included. Models were commonly co‐managed between surgical and geriatric teams, implemented at varying surgical pathway points. Orthopaedics represented 57% of studies. Geriatricians were involved in 90% of models; 38% included advanced practice nurses or specialist gerontological nurses. Comprehensive Geriatric Assessment was used in nearly half the studies, typically preoperatively. Considerable heterogeneity existed in model design, professional roles and care settings.

**Conclusion:**

Integrated geriatric perioperative care is expanding globally but remains limited outside orthopaedics. Research should shift from improvement projects to rigorous implementation for sustainable transformation, including nurse‐led models. Critical examination is needed of whether current outcomes address comprehensive needs of older surgical patients or primarily optimise hospital flow.

**Implications for the Profession and/or Patient Care:**

Findings highlight opportunities to expand geriatric models beyond orthopaedics and enhance nursing roles, particularly advanced practice nurses, in delivering comprehensive perioperative care for older adults.

**Impact:**

Addressed the gap in understanding how geriatric models of surgical care are operationalised. Identified underutilisation of nursing expertise and limited expansion beyond orthopaedics. Will impact service design, policy development and clinical implementation for older surgical patients.

**Reporting Method:**

Adhered to PRISMA Extension for Scoping Reviews (PRISMA‐ScR) guidelines.

**Patient or Public Contribution:**

No patient or public involvement.

**Protocol Registration:**

Open Science Framework Registries Network.

## What Does This Paper Contribute to the Wider Global Clinical Community?

- Maps core components of perioperative geriatric models to guide service design and implementation.
- Highlights the pivotal yet underutilised role of advanced practice nurses in geriatric surgical care.
- Identifies critical gaps in geriatric surgical care beyond orthopaedics requiring urgent attention.

## 1 Introduction

Global population ageing is driving unprecedented demand for surgery in older adults (United Nations 2024; Australian Bureau of Statistics 2020). Despite the potential benefits of surgery, age‐related physiological decline, frailty and multimorbidity increase perioperative risks, contributing to higher complication rates, functional decline and prolonged recovery (Thu et al. 2021; Richards et al. 2021). This demographic shift challenges traditional surgical care models and necessitates integrated, multidisciplinary approaches tailored to the complex needs of older surgical patients (United Nations 2024; Australian Bureau of Statistics 2020; Richards et al. 2021).

One such innovation is the Proactive Care of Older People undergoing Surgery (POPS) service, first developed in the UK in 2003. POPS integrates geriatricians into the surgical pathway and uses preoperative Comprehensive Geriatric Assessment (CGA) to optimise older adults undergoing surgery (Braude et al. 2017). This model has improved outcomes including reduced perioperative complications and length of stay, and has since been adopted across the UK National Health Service for a variety of elective general, urology and vascular surgeries (Waring et al. 2023).

CGA, originally developed in the 1990s to guide community‐based care for older adults, is now widely recognised as the gold standard for assessing and managing older adults across multiple settings (Ellis et al. 2011; Tan et al. 2024). Its multidimensional approach—covering medical, functional, cognitive and social domains—enables the development of individualised care plans that support risk reduction, preoperative optimisation and postoperative rehabilitation (Partridge et al. 2014).

Over time, CGA has been embedded into a range of geriatric models of care (GMOC) aimed at improving the quality and safety of surgical care for older adults. These models often comprise diverse components, including varying team structures, leadership models and degrees of integration into surgical pathways. Understanding which components are essential—and how they are operationalised in practice—is critical to improving care design, informing policy and guiding clinical implementation.

Nurses have a pivotal role in perioperative care for older adults, providing continuous bedside assessment, early detection of complications and coordination of multidisciplinary care. Advanced practice nurses, including Nurse Practitioners and Clinical Nurse Specialists with gerontological expertise, are key contributors to comprehensive geriatric assessment, care coordination and patient advocacy in surgical settings (Irimia et al. 2022; Van Leendert et al. 2021; Coskun and Duygulu 2022; Ayoub et al. 2023). Yet despite their central position in surgical care delivery, the specific contributions of nurses within geriatric surgical models remain poorly characterised in a predominantly physician‐centric literature. With health services internationally now developing advanced practice nursing roles to meet the needs of ageing surgical populations, understanding how nursing expertise is integrated into GMOCs is both timely and essential for informing service design.

A scoping review methodology was selected as the most appropriate approach for this study based on JBI guidance (Peters et al. 2020; Munn et al. 2018). According to JBI, scoping reviews are indicated when the primary purpose is to identify and map the types of available evidence, clarify key concepts or definitions, examine how research is conducted and identify knowledge gaps—all of which align with our objectives (Munn et al. 2018). Unlike systematic reviews that seek to answer focused clinical questions or synthesise effectiveness data, our aim was to map the landscape of geriatric surgical care models, characterise their components and identify patterns in their design and implementation (Munn et al. 2018). The considerable heterogeneity anticipated in model structures, populations and outcome measures rendered meta‐analysis inappropriate and supported the choice of a scoping review approach. Furthermore, this review serves an exploratory purpose—identifying what models exist and how they are operationalised—rather than determining which model is most effective, which would require a different review approach (Peters et al. 2020).

Thus, the objective of this scoping review was to identify and describe the core components of perioperative GMOC delivered in hospital settings for older adults undergoing surgery, including team composition, governance structures (e.g., leadership and integration) and the role of nurses. This review aimed to inform future service design by clarifying how geriatric care models are operationalised and what elements may be most important for achieving safe, effective and person‐centred surgical care for older populations.

### 1.1 Review Question

What hospital‐based, multidisciplinary geriatric models of care have been implemented for older adults undergoing surgery, and what are their characteristics?

## 2 Methods

### 2.1 Protocol and Registration

This review was conducted in accordance with the JBI methodology for scoping reviews (Peters et al. 2020) and reporting following the PRISMA Extension for Scoping Reviews (PRISMA‐ScR) guidelines (Table S1) (Tricco et al. 2018). The protocol was registered with Open Science Framework Registries Network (https://doi.org/10.17605/OSF.IO/V9NPC).

### 2.2 Selection Criteria

#### 2.2.1 Types of Participants

Older adults aged 65 years or older who are undergoing elective or emergency surgical procedures.

#### 2.2.2 Concept

Hospital‐based geriatric models of care, defined as structured, multidisciplinary approaches that integrate geriatric principles (including but not limited to CGA, frailty screening, geriatric syndrome management and care coordination) into surgical pathways, and require multidisciplinary involvement of at least two distinct health disciplines collaborating in patient care (e.g., two different medical specialties, or one medical and one allied health or nursing discipline) (Jankovic et al. 2024).

#### 2.2.3 Context

Acute hospital settings, including emergency departments, preoperative clinics, surgical wards and perioperative care units.

#### 2.2.4 Types of Studies

Studies that developed a GMOC, described characteristics of GMOC, or assessed the effectiveness of GMOC were included. Original studies that used any types of quantitative study designs, qualitative study designs or mixed methods studies were included. Other types of publication, such as literature reviews, clinical guidelines, policy reports, non‐peer‐reviewed research reports and articles, conference papers or opinion papers were excluded. More broadly, grey literature was excluded due to typically insufficient reporting detail and screening feasibility constraints.

#### 2.2.5 Outcomes

We aimed to capture characteristics of the models (what disciplines were involved in this care model and what were their roles?); governance details (who is/are key player/s in these models? Where did these models intersect? Where did these models sit within the surgical discipline: standing existing models (for every patient who meets certain criteria, there is a standard procedure/process to have other disciplines involved) within the surgical team, or invited role “by referral when needed,” or external to the surgical team? How did these ‘other’ disciplines get involved in these models of care: by invitation or already planned?); and care outcomes (including hospital and patient‐related outcomes).

### 2.3 Search Strategy

To ensure relevance to contemporary clinical practice and healthcare delivery models, the search strategy targeted original published studies in English between 1 January 2015 and 23 February 2025. A preliminary search spanning the last 20 years yielded an unmanageable number of results. Consequently, the review team narrowed the time frame to ensure feasibility while meeting the study objectives. An initial iterative search was undertaken in CINAHL and MEDLINE focusing on four primary conceptual domains: *geriatrics*, *models of care*, *surgery* and *hospital settings*. This process helped refine key terms and Medical Subject Headings (MESH). The full comprehensive search strategy can be found in Table S2. The search strategy was adapted to each of the six selected databases (CINAHL, MEDLINE, Embase, Scopus, AgeLine and Cochrane Library), including alterations to MeSH terms, truncations and search structures. This combination was comprehensive and selected to capture the interprofessional nature of geriatric surgical care. Final search results were exported to EndNote 20 (Clarivate) for reference management and duplicate removal. Records were then uploaded to Covidence systematic review software (Veritas Health Innovation, Melbourne, Australia) for team screening and review.

### 2.4 Study Selection Process

Title and abstract screening were performed independently by two reviewers (SH and UB, or SH and AT) using the PCC (Population, Concept, Context) selection criteria. Any conflicts were resolved through discussion with a third reviewer (CD). Full‐text screening of potentially eligible articles followed the same process, with two reviewers working independently and discrepancies adjudicated by a third.

### 2.5 Data Extraction and Charting Process

A data charting template was used for extraction of relevant information: bibliographic information (authors, year, country); study characteristics (design, sample size, setting); population details (where intervention commenced, surgical specialty); model components (team composition, roles, governance structure, interventions); and reported outcomes (see Table S3 for complete charting of intervention components). Extracted data were entered into Microsoft Excel and analysed descriptively. Frequencies and percentages were calculated to describe patterns in study characteristics, GMOC structures, roles and outcomes.

## 3 Results

### 3.1 Selection of Sources of Evidence

**FIGURE 1:**
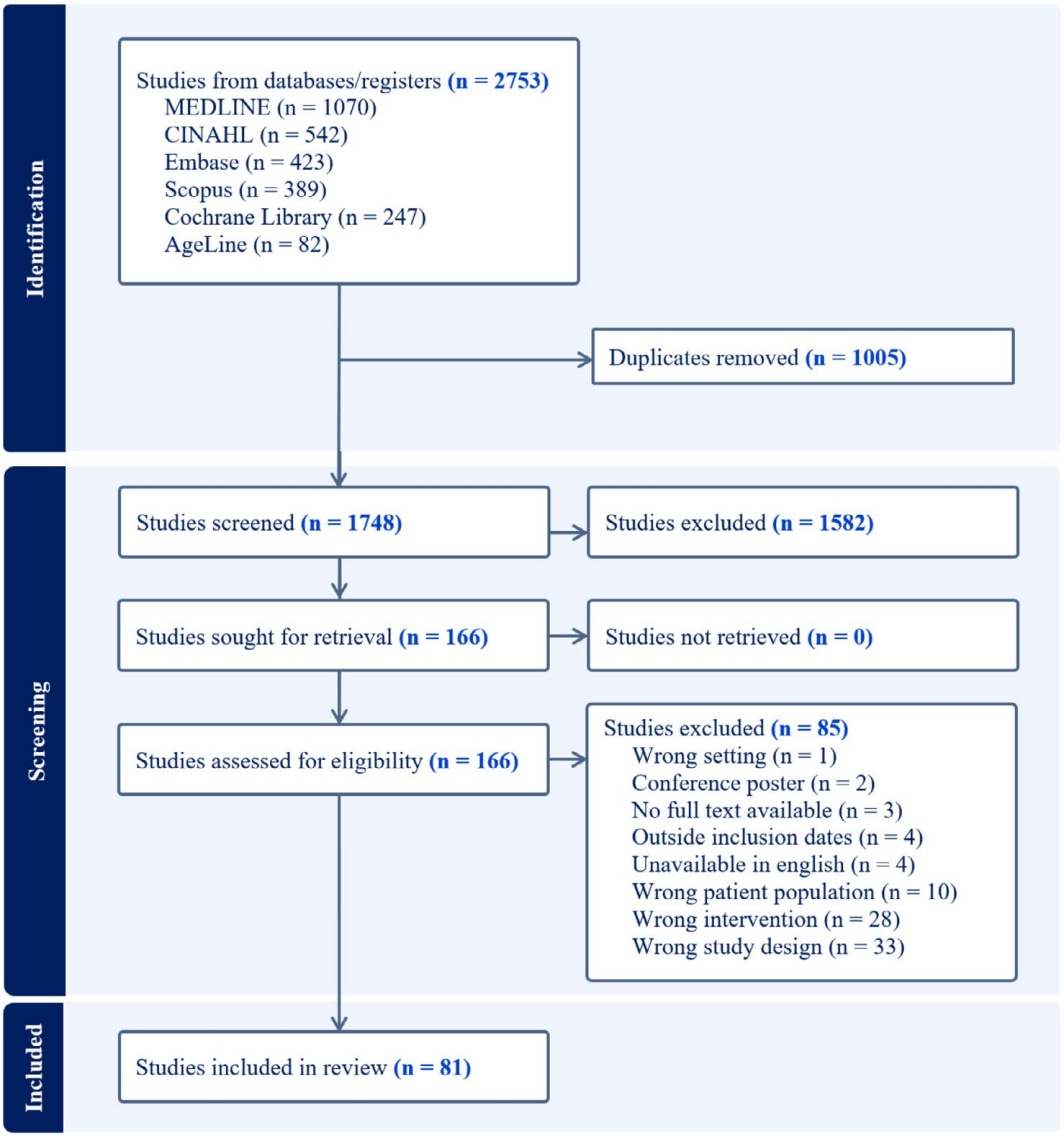
PRISMA flowchart of screen and selection (Tricco et al. 2018).

A total of 2753 studies were imported for screening, with 1005 duplicates removed (Figure 1). Title and abstract screening of 1748 articles was conducted independently by two reviewers, with 137 conflicts (7.8%) resolved by a third. Full‐text screening of 166 articles followed the same process, with discrepancies again resolved by a third reviewer. Eighty‐one studies were included for data extraction.

### 3.2 Study Characteristics

The 81 included studies represented a diverse methodological and geographic spread. Most originated from high‐income countries, with the US contributing the largest proportion (19%) (Adogwa et al. 2018; Breda et al. 2023; Chebl et al. 2024; Cizginer et al. 2023; Hu et al. 2022; Kregel et al. 2024; Lamb et al. 2017; Leede et al. 2020; Lester et al. 2022; Lin et al. 2021; McDonald et al. 2018; Neupane et al. 2023; Noticewala et al. 2016; Shahrokni et al. 2020; Wallace et al. 2019), followed by the UK (11%) (Braude et al. 2017; Checa‐López et al. 2024; de Las Casas et al. 2021; Irimia et al. 2022; Lisk et al. 2023; Middleton et al. 2017; Partridge et al. 2017, 2021; Shipway et al. 2018) and The Netherlands (10%) (Bremen et al. 2024; Festen et al. 2021; Kusen et al. 2021, 2022; Meulenbroek et al. 2024; Nijmeijer et al. 2018; van der Vlies et al. 2020; Van Leendert et al. 2021) (Figure S1). Three studies were conducted across multiple countries (Checa‐López et al. 2024; Kusen et al. 2022; Thillainadesan et al. 2021). Notably, studies from The Netherlands (Bremen et al. 2024; Festen et al. 2021; Kusen et al. 2021, 2022; Meulenbroek et al. 2024; Nijmeijer et al. 2018; van der Vlies et al. 2020; Van Leendert et al. 2021) and China (Liang et al. 2023; Liu et al. 2022, 2024; Peng et al. 2020; Wu et al. 2023; Xu et al. 2024; Zhou et al. 2024) did not emerge until later in the review period, first appearing in 2018 and 2020 respectively.

Methodologically, most studies were observational cohort studies: retrospective 42% (*n* = 34) (Adogwa et al. 2018; Kregel et al. 2024; Lamb et al. 2017; Leede et al. 2020; Neupane et al. 2023; Noticewala et al. 2016; Shahrokni et al. 2020; Middleton et al. 2017; Festen et al. 2021; Kusen et al. 2021, 2022, 2019; Nijmeijer et al. 2018; van der Vlies et al. 2020; Liang et al. 2023; Liu et al. 2022, 2024; Zhou et al. 2024; De Bueck et al. 2024; Forni et al. 2016; Higashikawa et al. 2022; Lau et al. 2017; Lynch et al. 2015; Neuerburg et al. 2019; Ogawa et al. 2022; Pablos‐Hernández et al. 2020; Pankratz et al. 2023; Shigemoto et al. 2022; Sura‐amonrattana et al. 2021; Tarazona‐Santabalbina et al. 2019; Werner et al. 2020; Baroni et al. 2019; Lee et al. 2021; Morris et al. 2023), prospective 11% (*n* = 9) (Hu et al. 2022; Lin et al. 2021; McDonald et al. 2018; Xu et al. 2024; Asplin et al. 2017; Henderson et al. 2017; Sim et al. 2023; Solberg et al. 2023; Thillainadesan et al. 2022), before‐and‐after 19% (*n* = 15) (Thu et al. 2021; Braude et al. 2017; Cizginer et al. 2023; Lester et al. 2022; Shipway et al. 2018; Duaso et al. 2018; Hofmeister et al. 2020; Murphy et al. 2019; Soong et al. 2016; Natesan et al. 2022; Wang et al. 2023; Khadaroo et al. 2020; Giannotti et al. 2022; Owen et al. 2023) and other cohort/modelling studies 9% (*n* = 7) (Breda et al. 2023; Chebl et al. 2024; Meulenbroek et al. 2024; Thillainadesan et al. 2021; Peng et al. 2020; Ramírez‐Martín et al. 2022; Murphy et al. 2019). Randomised controlled trials comprised 11% (*n* = 9) (Checa‐López et al. 2024; Partridge et al. 2017, 2021; Van Leendert et al. 2021; Wu et al. 2023; Coskun and Duygulu 2022; Gilbert et al. 2021; Thingstad et al. 2016; Tseng et al. 2019, 2021) while cross‐sectional 5% (*n* = 4) (Lisk et al. 2023; Bremen et al. 2024; Fagard et al. 2023; Solberg et al. 2023) and mixed‐methods studies 4% (*n* = 3) (de Las Casas et al. 2021; Mistry et al. 2023; Ayoub et al. 2023) were also represented. Sample sizes ranged from 45 to 23,973 participants (Forni et al. 2016; Ayoub et al. 2023).

**FIGURE 2:**
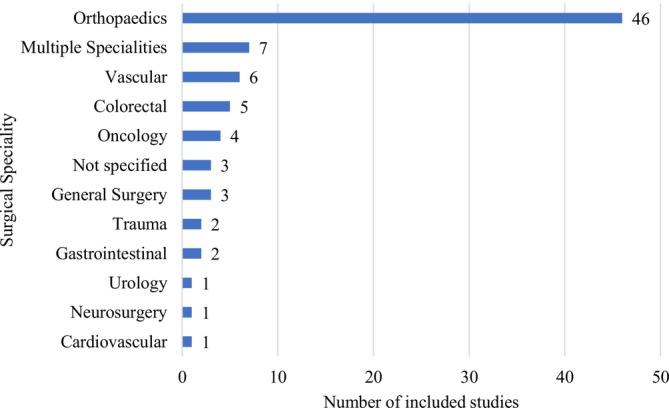
Surgical specialties represented in included studies.

Orthopaedic surgery was the most frequently represented speciality, accounting for 57% of studies, and was the only specialty reported consistently across the review period (Figure 2) (Breda et al. 2023; Lamb et al. 2017; Lin et al. 2021; Noticewala et al. 2016; Wallace et al. 2019; Lisk et al. 2023; Middleton et al. 2017; Bremen et al. 2024; Kusen et al. 2021, 2022, 2019; Nijmeijer et al. 2018; Van Leendert et al. 2021; Liang et al. 2023; Liu et al. 2022, 2024; Peng et al. 2020; Wu et al. 2023; Xu et al. 2024; Zhou et al. 2024; De Bueck et al. 2024; Forni et al. 2016; Higashikawa et al. 2022; Lau et al. 2017; Lynch et al. 2015; Neuerburg et al. 2019; Ogawa et al. 2022; Pablos‐Hernández et al. 2020; Pankratz et al. 2023; Shigemoto et al. 2022; Sura‐amonrattana et al. 2021; Werner et al. 2020; Mistry et al. 2023; Asplin et al. 2017; Henderson et al. 2017; Solberg et al. 2023; Thingstad et al. 2016; Tseng et al. 2019, 2021; Ayoub et al. 2023; Duaso et al. 2018; Murphy et al. 2019; Soong et al. 2016; Baroni et al. 2019; Lee et al. 2021). Other represented specialities included multiple speciality (9%) (Thu et al. 2021; Lester et al. 2022; McDonald et al. 2018; Neupane et al. 2023; de Las Casas et al. 2021; Thillainadesan et al. 2021; Fagard et al. 2023), vascular (7%) (Partridge et al. 2017, 2021; Meulenbroek et al. 2024; Thillainadesan et al. 2022; Natesan et al. 2022; Wang et al. 2023) and colorectal surgery (6%) (Cizginer et al. 2023; van der Vlies et al. 2020; Tarazona‐Santabalbina et al. 2019; Ramírez‐Martín et al. 2022; Gilbert et al. 2021).

### 3.3 Multidisciplinary Team

**FIGURE 3:**
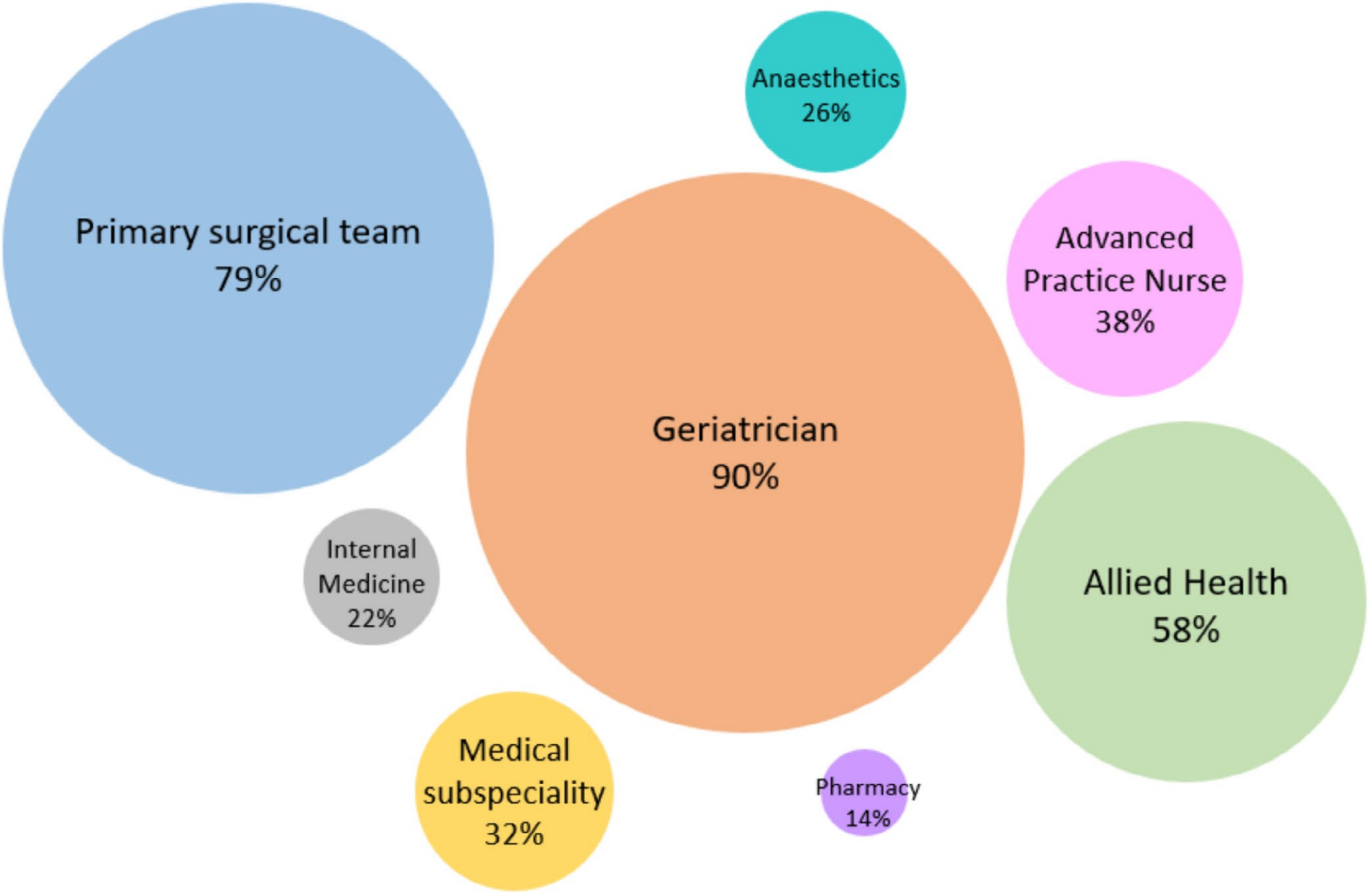
Disciplines represented in multidisciplinary geriatric surgical models.

The composition of multidisciplinary team members is illustrated in Figure 3, with roles outlined in Table 1. The surgical team was primarily responsible for operative care, ward rounds and multidisciplinary team (MDT) meetings. Geriatricians were included in 90% (*n =* 73) of studies, comprising both consultants and trainees. Their roles included conducting CGA, identifying and optimising risk factors, frailty and geriatric syndromes, as well as managing postoperative care and comorbidities. They frequently participated in ward rounds and MDT discussions and contributed to the education of medical and nursing staff on geriatric‐specific factors (Thu et al. 2021; Braude et al. 2017; Adogwa et al. 2018; Breda et al. 2023; Chebl et al. 2024; Cizginer et al. 2023; Hu et al. 2022; Lester et al. 2022; Lin et al. 2021; McDonald et al. 2018; Neupane et al. 2023; Noticewala et al. 2016; Shahrokni et al. 2020; Wallace et al. 2019; Checa‐López et al. 2024; de Las Casas et al. 2021; Irimia et al. 2022; Lisk et al. 2023; Partridge et al. 2017, 2021; Shipway et al. 2018; Bremen et al. 2024; Festen et al. 2021; Kusen et al. 2022, 2019; Meulenbroek et al. 2024; Nijmeijer et al. 2018; van der Vlies et al. 2020; Van Leendert et al. 2021; Thillainadesan et al. 2021, 2022; Liang et al. 2023; Liu et al. 2022, 2024; Peng et al. 2020; Xu et al. 2024; Zhou et al. 2024; De Bueck et al. 2024; Forni et al. 2016; Higashikawa et al. 2022; Lynch et al. 2015; Neuerburg et al. 2019; Ogawa et al. 2022; Pablos‐Hernández et al. 2020; Pankratz et al. 2023; Shigemoto et al. 2022; Sura‐amonrattana et al. 2021; Tarazona‐Santabalbina et al. 2019; Werner et al. 2020; Fagard et al. 2023; Mistry et al. 2023; Henderson et al. 2017; Ramírez‐Martín et al. 2022; Solberg et al. 2023; Gilbert et al. 2021; Thingstad et al. 2016; Tseng et al. 2019, 2021; Duaso et al. 2018; Hofmeister et al. 2020; Murphy et al. 2019; Soong et al. 2016; Baroni et al. 2019; Lee et al. 2021; Natesan et al. 2022; Wang et al. 2023; Khadaroo et al. 2020; Giannotti et al. 2022; Owen et al. 2023). Internal medicine was represented in 22% (*n =* 18) of studies, including hospitalists and acute physicians who worked closely with geriatricians on medical management (Hu et al. 2022; Lin et al. 2021; Noticewala et al. 2016; Middleton et al. 2017; Shipway et al. 2018; Kusen et al. 2022; Nijmeijer et al. 2018; Lau et al. 2017; Fagard et al. 2023; Sim et al. 2023; Coskun and Duygulu 2022; Tseng et al. 2019, 2021; Ayoub et al. 2023; Duaso et al. 2018; Soong et al. 2016; Owen et al. 2023; Morris et al. 2023). Medical subspecialists were included in 32% (*n = 26*) of studies, including cardiologists, endocrinologists, oncologists, psychiatrists and critical care and emergency physicians (Thu et al. 2021; Cizginer et al. 2023; Lamb et al. 2017; Lester et al. 2022; Neupane et al. 2023; Shahrokni et al. 2020; Checa‐López et al. 2024; Festen et al. 2021; van der Vlies et al. 2020; Liang et al. 2023; Liu et al. 2024; Peng et al. 2020; Wu et al. 2023; Xu et al. 2024; Zhou et al. 2024; Shigemoto et al. 2022; Henderson et al. 2017; Ramírez‐Martín et al. 2022; Sim et al. 2023; Murphy et al. 2019; Kusen et al. 2019). Allied health roles were described in 58% (*n = 47*) of studies and included physiotherapists, occupational therapists, dieticians and social workers (Thu et al. 2021; Braude et al. 2017; Adogwa et al. 2018; Chebl et al. 2024; Lamb et al. 2017; Lester et al. 2022; Noticewala et al. 2016; Wallace et al. 2019; Checa‐López et al. 2024; de Las Casas et al. 2021; Irimia et al. 2022; Lisk et al. 2023; Partridge et al. 2017, 2021; Shipway et al. 2018; Kusen et al. 2022, 2019; Nijmeijer et al. 2018; van der Vlies et al. 2020; Liang et al. 2023; Liu et al. 2022, 2024; Peng et al. 2020; Wu et al. 2023; Xu et al. 2024; Zhou et al. 2024; De Bueck et al. 2024; Lau et al. 2017; Neuerburg et al. 2019; Pablos‐Hernández et al. 2020; Pankratz et al. 2023; Shigemoto et al. 2022; Sura‐amonrattana et al. 2021; Tarazona‐Santabalbina et al. 2019; Asplin et al. 2017; Sim et al. 2023; Solberg et al. 2023; Coskun and Duygulu 2022; Gilbert et al. 2021; Thingstad et al. 2016; Tseng et al. 2019; Murphy et al. 2019; Soong et al. 2016; Giannotti et al. 2022).

**TABLE 1:** Multidisciplinary roles in geriatric surgical models of care.

Role	No. of studies	Role description
Primary surgical team	64	Surgical management of patients Daily ward round and weekly MDT meetings
Geriatricians	73	Comprehensive geriatric assessments Identification and optimisation of risks Post operative care and management of comorbidities and post operative complications in particular delirium, frailty and pain Participation in daily ward rounds and MDT meetings Training medical, surgical and nursing staff Management of polypharmacy
Internal medicine	18	Medical management as directed by geriatrician Assessment and management of patients outside geriatrician hours
Medical subspeciality	26	Speciality specific care as required via referral
Anaesthetics	21	Preoperative evaluation Address anaesthetic‐related issues Post operative pain management
Allied health	47	Post operative mobility and rehabilitation Nutritional support Discharge planning
Pharmacy	11	Medication management, polypharmacy advice, deprescribing inappropriate medications

Advanced practice nurse or specialist gerontological nurse roles were identified in 38% (*n =* 31) of studies. This included roles specified as Nurse Practitioner, Clinical Nurse Specialist, or those referred to as ‘geriatric nurse’ without further detail as seen in 31 studies (Table 2). Three studies described Nurse Practitioner roles that were not geriatric specific, instead focusing on trauma, emergency or acute surgical care (Thu et al. 2021; Nijmeijer et al. 2018; Murphy et al. 2019). The term “Clinical Nurse Specialist” was first reported in two studies published in 2017 (Braude et al. 2017; Partridge et al. 2017), while the first mention of Nurse Practitioners occurred in 2020 (Breda et al. 2023; Kregel et al. 2024; Leede et al. 2020; Lin et al. 2021; Irimia et al. 2022; Van Leendert et al. 2021; Ayoub et al. 2023; Lee et al. 2021).

**TABLE 2:** Advanced practice nurse roles in geriatric surgical models of care.

Role	No. of studies	Surgical speciality	Role description
Geriatric nurse	14	Colorectal (Tarazona‐Santabalbina et al. 2019; Ramírez‐Martín et al. 2022) General surgery (Khadaroo et al. 2020) Multiple specialities (Lester et al. 2022; McDonald et al. 2018) Orthopaedics (De Bueck et al. 2024; Mistry et al. 2023; Tseng et al. 2019, 2021) Not specified (Checa‐López et al. 2024; Festen et al. 2021; Fagard et al. 2023; Coskun and Duygulu 2022; Owen et al. 2023)	Comprehensive geriatric assessment Daily follow up Discharge planning Implementation of care plan established with a geriatrician and evidence‐based practices
Nurse practitioner	11	Orthopaedics (Breda et al. 2023; Lin et al. 2021; Nijmeijer et al. 2018; Van Leendert et al. 2021; Lau et al. 2017; Ayoub et al. 2023; Murphy et al. 2019) Trauma (Kregel et al. 2024; Leede et al. 2020) Multiple specialties (Thu et al. 2021; Irimia et al. 2022)	Comprehensive geriatric assessment Diagnosis and management of comorbidities and postoperative complications Initiation of interventions e.g., referrals for investigations, medical specialties and allied health Medication/polypharmacy management Discharge planning Patient and family education Daily rounds and MDT meetings Hospital staff development
Clinical nurse specialist	3	Urology (Braude et al. 2017) Vascular (Partridge et al. 2017; Thillainadesan et al. 2022)	Comprehensive geriatric assessment Daily rounds MDT meetings
Nurse navigator	1	Oncology (Chebl et al. 2024)	Preoperative assessments Optimise postoperative care Initiation of interventions e.g., referrals for investigations, medical specialties and allied health Patient education Discharge planning
Advanced practice nurse	2	Vascular (Natesan et al. 2022) Multiple specialities (Fagard et al. 2023)	Comprehensive geriatric assessment Optimisation of comorbidity treatments Address complications Discharge planning

Advanced practice or gerontologic nursing roles were reported across all surgical specialities included in the review, except for neurosurgery. Orthopaedic surgery remained the most represented speciality, with 35% (*n =* 11) of these studies involving either geriatric specialisation (*n =* 4) or Nurse Practitioner (*n =* 7) roles (Breda et al. 2023; Lin et al. 2021; Nijmeijer et al. 2018; Van Leendert et al. 2021; Ayoub et al. 2023; Murphy et al. 2019; Lee et al. 2021). Among countries represented in the review, 59% explicitly identified a geriatric or advanced practice nursing role in their described model of care.

All reported roles included the completion of CGAs as a core responsibility. Nurse Practitioners were described as functioning autonomously, performing CGAs, identifying and managing comorbidities and geriatric syndromes, initiating investigations and referrals, prescribing medications and providing patients and family education and support (Table 2) (Thu et al. 2021; Breda et al. 2023; Kregel et al. 2024; Leede et al. 2020; Lin et al. 2021; Irimia et al. 2022; Nijmeijer et al. 2018; Van Leendert et al. 2021; Lau et al. 2017; Ayoub et al. 2023; Murphy et al. 2019). In models that included Nurse Practitioners (*n* = 11), most were led by geriatrics or medical teams, external to the surgical team. These models described Nurse Practitioners contributing to perioperative care through clinical, diagnostic and supportive roles.

### 3.4 Characteristics of the Models of Care

#### 3.4.1 Model Structures and Entry Points

Traditional models of care involved surgical team‐led management, with reactive, referral‐based medical input. In contrast, four predominant models of geriatric surgical care were identified, distinguished by their referral mechanisms and leadership structures (Table 3).

**TABLE 3:** Typology of geriatric surgical care models by referral method and leadership.

Model of care	No. of studies	Setting	Integration of geriatrician or advanced practice nurse	Leadership
Geriatric‐specific surgical ward or dedicated geriatric beds on a surgical ward (Kregel et al. 2024; Lamb et al. 2017; Lisk et al. 2023; Middleton et al. 2017; Kusen et al. 2021, 2022; Nijmeijer et al. 2018; Liu et al. 2022, 2024; Peng et al. 2020; Xu et al. 2024; Neuerburg et al. 2019; Pablos‐Hernández et al. 2020; Asplin et al. 2017; Solberg et al. 2023; Gilbert et al. 2021; Thingstad et al. 2016; Duaso et al. 2018; Khadaroo et al. 2020)	20	Admission, inpatient surgical ward	Geriatrician integrated into ward team, shared decision‐making in‐patient care during hospitalisation period	Collaborative shared leadership model by surgical team and geriatric specialist
Screening based referral for geriatric involvement (Hu et al. 2022; Kregel et al. 2024; Lin et al. 2021; McDonald et al. 2018; Neupane et al. 2023; Shahrokni et al. 2020; Wallace et al. 2019; Shipway et al. 2018; Meulenbroek et al. 2024; Nijmeijer et al. 2018; van der Vlies et al. 2020; Ogawa et al. 2022; Pablos‐Hernández et al. 2020; Pankratz et al. 2023; Werner et al. 2020; Solberg et al. 2023; Tseng et al. 2019; Kusen et al. 2019; Natesan et al. 2022)	19	Emergency department, inpatient surgical ward, preoperative clinic	Assessment and screening by non‐geriatric specialist (e.g., emergency physician, anaesthetist, surgical team) referral made for gerontological input if specified criteria met	Surgical team
Targeted case finding by geriatric specialist (Braude et al. 2017; Chebl et al. 2024; Leede et al. 2020; de Las Casas et al. 2021; Middleton et al. 2017; van der Vlies et al. 2020; Liang et al. 2023; Giannotti et al. 2022; Morris et al. 2023)	8	Emergency department, inpatient surgical ward	Geriatric specialist attends emergency department or MDT meetings to identify patients requiring gerontological management	Geriatric specialists
Blanket referral for anyone ≥ 65 years (Thu et al. 2021; Cizginer et al. 2023; Leede et al. 2020; Noticewala et al. 2016; Checa‐López et al. 2024; Irimia et al. 2022; Partridge et al. 2017, 2021; Festen et al. 2021; Xu et al. 2024; Zhou et al. 2024; De Bueck et al. 2024; Tarazona‐Santabalbina et al. 2019; Henderson et al. 2017; Ramírez‐Martín et al. 2022; Sim et al. 2023; Gilbert et al. 2021; Ayoub et al. 2023; Murphy et al. 2019; Soong et al. 2016; Baroni et al. 2019; Lee et al. 2021; Wang et al. 2023; Owen et al. 2023)	25	Preoperative clinic, emergency department, inpatient surgical ward	Geriatric involvement for all patients > 65 years of age, co‐management of patients across the surgical journey	Comanaged by geriatric specialist and surgical team

*Geriatric‐specific surgical wards or dedicated beds* (*n* = 20 studies) represent the most integrated approach, where geriatricians are embedded within surgical ward teams and participate in shared decision‐making throughout hospitalisation. These models typically involve daily geriatric rounds and joint responsibility for patient care during hospitalisation.

*Screening‐based referral models* (*n* = 19 studies) employ initial assessment by non‐geriatric specialists (emergency physicians, anaesthetists or surgical teams) who refer patients meeting specified criteria (e.g., age thresholds, frailty scores, cognitive impairment) for geriatric consultation. Leadership typically remains with the surgical team.

*Targeted case‐finding models* (*n* = 8 studies) position geriatric specialists to proactively identify patients requiring intervention through attendance at emergency departments or multidisciplinary team meetings, rather than awaiting referrals. These models are led by geriatric specialists who actively seek patients who would benefit from their expertise.

*Blanket referral models* (*n* = 25 studies) provide automatic geriatric involvement for all patients meeting age criteria (typically ≥ 65 years), enabling comprehensive co‐management across the surgical journey. These models represent shared leadership between geriatric and surgical teams and were the most frequently reported approach.

Geriatric availability varied widely across all model types—some provided daily or twice‐weekly rounds, others were limited to office hours or preoperative input only—reflecting workforce variation across health systems. For example, Tarazona‐Santabalbina et al. (Tarazona‐Santabalbina et al. 2019) described continuous geriatric involvement throughout the surgical journey, while other models included comprehensive preoperative assessment by geriatricians or clinical nurse specialists, who developed postoperative care plans followed by surgical team delivery.

Intervention commenced at different points: emergency department, surgical admission, postoperative period or preoperative clinic. Six studies reported multi‐point initiation (Wallace et al. 2019; Lisk et al. 2023; Thillainadesan et al. 2021; Peng et al. 2020; Fagard et al. 2023; Solberg et al. 2023). Among orthopaedic models, 47% (*n* = 23) began in the emergency department (Figure S2), reflecting acute fracture presentations. When orthopaedic studies were excluded, 49% (*n =* 18) of remaining models commenced in the preoperative clinic (Adogwa et al. 2018; Chebl et al. 2024; Lester et al. 2022; McDonald et al. 2018; Shahrokni et al. 2020; de Las Casas et al. 2021; Partridge et al. 2017, 2021; Shipway et al. 2018; Festen et al. 2021; Meulenbroek et al. 2024; van der Vlies et al. 2020; Thillainadesan et al. 2021; Fagard et al. 2023; Ramírez‐Martín et al. 2022; Sim et al. 2023; Gilbert et al. 2021; Giannotti et al. 2022) and only 14% (*n =* 5) in the emergency department (Figure S3), reflecting opportunities for proactive assessment in elective surgeries (Checa‐López et al. 2024; Thillainadesan et al. 2021; Fagard et al. 2023; Hofmeister et al. 2020; Owen et al. 2023).

#### 3.4.2 Assessment and Intervention Components

Geriatric assessment was described in 75% (*n =* 61) of studies, with CGA adopted in 46% (*n* = 37) (Thu et al. 2021; Breda et al. 2023; Chebl et al. 2024; Cizginer et al. 2023; Hu et al. 2022; Kregel et al. 2024; Lin et al. 2021; Neupane et al. 2023; Checa‐López et al. 2024; de Las Casas et al. 2021; Irimia et al. 2022; Partridge et al. 2017, 2021; Shipway et al. 2018; Bremen et al. 2024; Kusen et al. 2021, 2022, 2019; Meulenbroek et al. 2024; Nijmeijer et al. 2018; van der Vlies et al. 2020; Van Leendert et al. 2021; Thillainadesan et al. 2021, 2022; De Bueck et al. 2024; Pablos‐Hernández et al. 2020; Tarazona‐Santabalbina et al. 2019; Mistry et al. 2023; Henderson et al. 2017; Ramírez‐Martín et al. 2022; Tseng et al. 2021; Soong et al. 2016; Baroni et al. 2019; Lee et al. 2021; Natesan et al. 2022; Wang et al. 2023; Giannotti et al. 2022). Other studies used modified or domain‐specific assessments to support perioperative planning. All GMOC intervention components across studies were charted in Table S3. Pain management strategies were common and tailored to older adults with cognitive impairment or multimorbidity (Thu et al. 2021; Chebl et al. 2024; Cizginer et al. 2023; Kregel et al. 2024; Lamb et al. 2017; Lester et al. 2022; Lin et al. 2021; McDonald et al. 2018; Shahrokni et al. 2020; Wallace et al. 2019; Liu et al. 2024; Wu et al. 2023; Zhou et al. 2024; Sura‐amonrattana et al. 2021; Tarazona‐Santabalbina et al. 2019; Sim et al. 2023; Thillainadesan et al. 2022; Ayoub et al. 2023; Duaso et al. 2018; Murphy et al. 2019; Soong et al. 2016; Natesan et al. 2022; Giannotti et al. 2022). Nutrition and fluid optimisation were also frequently addressed through dietitian input, screening tools, supplementation (Thu et al. 2021; Chebl et al. 2024; Lamb et al. 2017; Lester et al. 2022; McDonald et al. 2018; Wallace et al. 2019; Checa‐López et al. 2024; Festen et al. 2021; Meulenbroek et al. 2024; Nijmeijer et al. 2018; Van Leendert et al. 2021; Liu et al. 2022, 2024; Zhou et al. 2024; Pankratz et al. 2023; Shigemoto et al. 2022; Sura‐amonrattana et al. 2021; Tarazona‐Santabalbina et al. 2019; Ramírez‐Martín et al. 2022; Sim et al. 2023; Thillainadesan et al. 2022; Coskun and Duygulu 2022; Gilbert et al. 2021; Kusen et al. 2019; Natesan et al. 2022; Giannotti et al. 2022). Some models prioritised surgical intervention to prevent deterioration, while early mobilisation was used across specialities to reduce postoperative complications and recovery (Breda et al. 2023; Lin et al. 2021; Lisk et al. 2023; Middleton et al. 2017; Kusen et al. 2021, 2022, 2019; Nijmeijer et al. 2018; Liang et al. 2023; Peng et al. 2020; Wu et al. 2023; Zhou et al. 2024; Sura‐amonrattana et al. 2021; Werner et al. 2020; Solberg et al. 2023). Discharge planning and coordinated transition to post‐acute services were widely reported (Thu et al. 2021; Breda et al. 2023; Chebl et al. 2024; Cizginer et al. 2023; Hu et al. 2022; Leede et al. 2020; Lester et al. 2022; Lin et al. 2021; McDonald et al. 2018; Shahrokni et al. 2020; Wallace et al. 2019; Checa‐López et al. 2024; de Las Casas et al. 2021; Irimia et al. 2022; Partridge et al. 2021; Shipway et al. 2018; Kusen et al. 2021, 2022; Nijmeijer et al. 2018; van der Vlies et al. 2020; Van Leendert et al. 2021; Liu et al. 2022; Wu et al. 2023; Forni et al. 2016; Lynch et al. 2015; Pankratz et al. 2023; Sura‐amonrattana et al. 2021; Tarazona‐Santabalbina et al. 2019; Asplin et al. 2017; Thillainadesan et al. 2022; Thingstad et al. 2016; Tseng et al. 2019, 2021; Ayoub et al. 2023; Duaso et al. 2018; Soong et al. 2016; Baroni et al. 2019; Natesan et al. 2022; Wang et al. 2023; Khadaroo et al. 2020; Morris et al. 2023) with 10 studies describing ongoing geriatric follow‐up up to 6 months post discharge (Breda et al. 2023; Chebl et al. 2024; Lester et al. 2022; Lin et al. 2021; Middleton et al. 2017; Xu et al. 2024; Ramírez‐Martín et al. 2022; Sim et al. 2023; Coskun and Duygulu 2022; Tseng et al. 2019). These models highlight the importance of extending care beyond hospitalisation to support sustained recovery.

#### 3.4.3 Leadership and Governance

Leadership was most frequently described as comanaged (65%, *n = 5*3) between the surgical team and geriatric teams (Adogwa et al. 2018; Chebl et al. 2024; Cizginer et al. 2023; Leede et al. 2020; Lester et al. 2022; Lin et al. 2021; McDonald et al. 2018; Noticewala et al. 2016; Lisk et al. 2023; Middleton et al. 2017; Bremen et al. 2024; Festen et al. 2021; Kusen et al. 2021, 2022, 2019; Nijmeijer et al. 2018; van der Vlies et al. 2020; Thillainadesan et al. 2021, 2022; Liu et al. 2022, 2024; Peng et al. 2020; Wu et al. 2023; Xu et al. 2024; Zhou et al. 2024; De Bueck et al. 2024; Forni et al. 2016; Higashikawa et al. 2022; Lau et al. 2017; Lynch et al. 2015; Neuerburg et al. 2019; Ogawa et al. 2022; Pablos‐Hernández et al. 2020; Pankratz et al. 2023; Shigemoto et al. 2022; Sura‐amonrattana et al. 2021; Tarazona‐Santabalbina et al. 2019; Fagard et al. 2023; Mistry et al. 2023; Henderson et al. 2017; Ramírez‐Martín et al. 2022; Solberg et al. 2023; Coskun and Duygulu 2022; Duaso et al. 2018; Hofmeister et al. 2020; Murphy et al. 2019; Soong et al. 2016; Baroni et al. 2019; Lee et al. 2021; Natesan et al. 2022; Wang et al. 2023; Khadaroo et al. 2020; Morris et al. 2023). Terms such as “comanaged,” “collaborative,” “integrated,” “shared care,” and “shared leadership,” were frequently used. In 31% (*n =* 25) of studies, care was led by geriatric or internal medicine specialists in close collaboration with surgical and multidisciplinary teams (Thu et al. 2021; Braude et al. 2017; Breda et al. 2023; Hu et al. 2022; Kregel et al. 2024; Lamb et al. 2017; Neupane et al. 2023; Shahrokni et al. 2020; Checa‐López et al. 2024; de Las Casas et al. 2021; Irimia et al. 2022; Partridge et al. 2017, 2021; Shipway et al. 2018; Meulenbroek et al. 2024; Van Leendert et al. 2021; Asplin et al. 2017; Sim et al. 2023; Gilbert et al. 2021; Thingstad et al. 2016; Tseng et al. 2019, 2021; Ayoub et al. 2023; Giannotti et al. 2022; Owen et al. 2023), with only 4% (*n =* 3) of models led solely by the surgical team (Wallace et al. 2019; Liang et al. 2023; Werner et al. 2020).

### 3.5 Outcomes

#### 3.5.1 Clinical Outcomes

Studies frequently reported on hospital length of stay (64%, *n =* 52) (Braude et al. 2017; Breda et al. 2023; Chebl et al. 2024; Cizginer et al. 2023; Kregel et al. 2024; Lamb et al. 2017; Leede et al. 2020; Lester et al. 2022; Lin et al. 2021; McDonald et al. 2018; Noticewala et al. 2016; Wallace et al. 2019; de Las Casas et al. 2021; Lisk et al. 2023; Middleton et al. 2017; Partridge et al. 2017, 2021; Shipway et al. 2018; Bremen et al. 2024; Festen et al. 2021; Kusen et al. 2021, 2022, 2019; Meulenbroek et al. 2024; Nijmeijer et al. 2018; Van Leendert et al. 2021; Liang et al. 2023; Liu et al. 2022; Wu et al. 2023; Xu et al. 2024; Zhou et al. 2024; Lau et al. 2017; Lynch et al. 2015; Ogawa et al. 2022; Pablos‐Hernández et al. 2020; Shigemoto et al. 2022; Sura‐amonrattana et al. 2021; Werner et al. 2020; Henderson et al. 2017; Ramírez‐Martín et al. 2022; Solberg et al. 2023; Thillainadesan et al. 2022; Gilbert et al. 2021; Ayoub et al. 2023; Duaso et al. 2018; Soong et al. 2016; Baroni et al. 2019; Lee et al. 2021; Natesan et al. 2022; Khadaroo et al. 2020; Giannotti et al. 2022; Owen et al. 2023) and mortality (62%, *n =* 50) (Thu et al. 2021; Braude et al. 2017; Breda et al. 2023; Cizginer et al. 2023; Kregel et al. 2024; Lamb et al. 2017; Leede et al. 2020; Lester et al. 2022; Neupane et al. 2023; Noticewala et al. 2016; Shahrokni et al. 2020; Wallace et al. 2019; Checa‐López et al. 2024; Middleton et al. 2017; Bremen et al. 2024; Festen et al. 2021; Kusen et al. 2021, 2022, 2019; Meulenbroek et al. 2024; Nijmeijer et al. 2018; van der Vlies et al. 2020; Van Leendert et al. 2021; Liang et al. 2023; Liu et al. 2022; Peng et al. 2020; Xu et al. 2024; Forni et al. 2016; Higashikawa et al. 2022; Lau et al. 2017; Neuerburg et al. 2019; Ogawa et al. 2022; Pablos‐Hernández et al. 2020; Pankratz et al. 2023; Shigemoto et al. 2022; Sura‐amonrattana et al. 2021; Tarazona‐Santabalbina et al. 2019; Werner et al. 2020; Mistry et al. 2023; Henderson et al. 2017; Ramírez‐Martín et al. 2022; Solberg et al. 2023; Tseng et al. 2019; Ayoub et al. 2023; Duaso et al. 2018; Soong et al. 2016; Baroni et al. 2019; Khadaroo et al. 2020; Giannotti et al. 2022; Morris et al. 2023), across varying follow‐up periods. Most studies observed a reduction in length of stay. Mortality outcomes were inconsistent, with no strong evidence of benefit.

Surgical complication rates were reported in 47% (*n =* 38) of studies, although definitions varied, including general categories (e.g., “postoperative complications”) and specific events such as urinary tract infections, deep vein thrombosis, pressure injuries, cardiac events, acute kidney injury, blood loss and rates of intensive care unit admission (Thu et al. 2021; Braude et al. 2017; Adogwa et al. 2018; Cizginer et al. 2023; Kregel et al. 2024; Lamb et al. 2017; Lester et al. 2022; McDonald et al. 2018; Noticewala et al. 2016; Partridge et al. 2017; Bremen et al. 2024; Festen et al. 2021; Kusen et al. 2021, 2022, 2019; Nijmeijer et al. 2018; van der Vlies et al. 2020; Van Leendert et al. 2021; Liang et al. 2023; Liu et al. 2022, 2024; Peng et al. 2020; Zhou et al. 2024; De Bueck et al. 2024; Lau et al. 2017; Pankratz et al. 2023; Shigemoto et al. 2022; Tarazona‐Santabalbina et al. 2019; Werner et al. 2020; Henderson et al. 2017; Ramírez‐Martín et al. 2022; Thillainadesan et al. 2022; Gilbert et al. 2021; Natesan et al. 2022; Khadaroo et al. 2020; Giannotti et al. 2022; Morris et al. 2023). Delirium was reported in 27% (*n* = 22) of studies (Thu et al. 2021; Adogwa et al. 2018; Chebl et al. 2024; Cizginer et al. 2023; Hu et al. 2022; Kregel et al. 2024; Lester et al. 2022; McDonald et al. 2018; Partridge et al. 2017; Kusen et al. 2022, 2019; Meulenbroek et al. 2024; Nijmeijer et al. 2018; Liang et al. 2023; De Bueck et al. 2024; Sura‐amonrattana et al. 2021; Tarazona‐Santabalbina et al. 2019; Ramírez‐Martín et al. 2022; Thillainadesan et al. 2022; Lee et al. 2021; Natesan et al. 2022; Khadaroo et al. 2020).

#### 3.5.2 Process and Economic Outcomes

Time to surgery was reported in 26% (*n =* 21) of studies, all in orthopaedics (Lin et al. 2021; Noticewala et al. 2016; Middleton et al. 2017; Bremen et al. 2024; Kusen et al. 2021; Nijmeijer et al. 2018; Liang et al. 2023; Liu et al. 2022; Xu et al. 2024; Zhou et al. 2024; Lynch et al. 2015; Ogawa et al. 2022; Pablos‐Hernández et al. 2020; Shigemoto et al. 2022; Sura‐amonrattana et al. 2021; Werner et al. 2020; Solberg et al. 2023; Murphy et al. 2019; Soong et al. 2016; Baroni et al. 2019; Lee et al. 2021). Discharge destination was reported in 17 studies, with mixed findings regarding discharge home versus alternative care settings (Cizginer et al. 2023; Kregel et al. 2024; Lamb et al. 2017; Leede et al. 2020; McDonald et al. 2018; Partridge et al. 2017; Van Leendert et al. 2021; Neuerburg et al. 2019; Ogawa et al. 2022; Pankratz et al. 2023; Mistry et al. 2023; Solberg et al. 2023; Duaso et al. 2018; Kusen et al. 2019; Lee et al. 2021; Khadaroo et al. 2020; Owen et al. 2023). Functional outcomes and Quality‐Adjusted Life Years were inconsistently reported across 11 studies (Checa‐López et al. 2024; Liu et al. 2022; Peng et al. 2020; Xu et al. 2024; Ogawa et al. 2022; Werner et al. 2020; Asplin et al. 2017; Henderson et al. 2017; Coskun and Duygulu 2022; Thingstad et al. 2016; Hofmeister et al. 2020), with wide variation in populations, surgical procedures and outcome measures. Cost‐effectiveness was evaluated in 12 studies, including metrics such as total costs, healthcare utilisation costs and workforce‐related expenditure (Breda et al. 2023; Cizginer et al. 2023; Lin et al. 2021; Lisk et al. 2023; Partridge et al. 2021; Peng et al. 2020; Xu et al. 2024; Lau et al. 2017; Shigemoto et al. 2022; Ayoub et al. 2023; Hofmeister et al. 2020; Soong et al. 2016).

## 4 Discussion

This scoping review highlights the global shift toward integrated models of care for older adults undergoing surgery, recognising their vulnerability from comorbidities and geriatric syndromes. Despite this, postoperative outcomes remain suboptimal, underscoring the need for system‐level innovation and proactive, collaborative care (Thu et al. 2021; Irimia et al. 2022; Thingstad et al. 2016).

Geriatric‐led co‐management models have traditionally centred on orthogeriatric models, which have set the standard for collaborative care (Thu et al. 2021; Lynch et al. 2015). A pivotal advancement came with the introduction of the POPS service in the UK, formalising a proactive, multidisciplinary approach anchored by CGA across the perioperative journey (Braude et al. 2017). However, proactive geriatric models remain under‐represented in specialties such as urology, colorectal, vascular and neurosurgery and in countries including Australia, highlighting opportunities for further research and development.

The CGA was the foundation for team intervention across the perioperative pathway in approximately half of the included studies. Unlike routine clinical assessments, CGA adopts a whole‐person approach, identifying medical, psychosocial and functional needs to guide coordinated, person‐centred care (Irimia et al. 2022; Partridge et al. 2021; Veronese et al. 2022; Miller et al. 2022). Evidence from RCTs and meta‐analyses shows that CGA improves surgical risk stratification (Chuang et al. 2022; Bao et al. 2023), mobilisation, functional recovery (Saripella et al. 2021), discharge planning (Saripella et al. 2021) and reduces readmissions (Van Grootven et al. 2018). Uptake in perioperative settings remains limited due to time demands, the need for multidisciplinary input and workforce shortages (Veronese et al. 2022; Miller et al. 2022). These challenges have prompted growing interest in modified CGA assessments that preserve diagnostic value while fitting into fast‐paced surgical workflows. Addressing these barriers is essential to expanding CGA access and improving perioperative outcomes. Additionally, the review identified limited attention to advance care planning and surgical wound complication prevention—two critical, yet underrepresented, elements of holistic perioperative care for older adults.

Gerontological specialty nurses—particularly advanced practice nurses such as Nurse Practitioners—when employed, were key contributors to successful implementation. These roles more readily identified geriatric syndromes, initiated timely interventions and supported advance care planning, functions traditionally performed by geriatricians. Their consistent presence on the ward, unlike rotating doctors, also enhanced continuity (Irimia et al. 2022; Van Leendert et al. 2021; Ayoub et al. 2023). Models where nurse specialists replaced or supplemented geriatricians showed positive outcomes in patient experience and multidisciplinary team collaboration. The inclusion of CGAs as a core function across nurse‐led roles highlights their capacity to deliver comprehensive geriatric care. Nurse Practitioners delivering CGA enabled timely risk stratification and tailored interventions, addressing system‐level barriers to comprehensive surgical optimisation.

Despite evidence supporting the implementation of integrated geriatric perioperative models, several barriers were identified. The most common was inadequate funding, particularly for staffing nurse‐led initiatives (Thillainadesan et al. 2021, 2022; Fagard et al. 2023; Mistry et al. 2023). Another was role ambiguity within multidisciplinary teams, especially concerns raised by geriatricians about overlapping responsibilities. Lack of clarity risked duplicating or undervaluing geriatricians' contributions and, in some cases, was perceived as encroachment by other specialties. Poorly defined roles were linked to conflict, inefficiency and threats to patient safety despite interprofessional models generally improving communication and team satisfaction.

A further barrier was the lack of formal recognition of perioperative geriatric programs, often embedded within existing pathways without distinct mandates or structures (Thillainadesan et al. 2021, 2022; Mistry et al. 2023; Ayoub et al. 2023). Workforce limitations, particularly the shortage of clinicians with perioperative geriatric expertise, were also prominent (Fagard et al. 2023; Mistry et al. 2023; Thillainadesan et al. 2022; Ayoub et al. 2023). These barriers underscore the need for clear role definitions, formalised structures to support sustainable, effective models of care.

Several facilitators supported the successful implementation of geriatric models in surgical settings. At the organisational level, strong nursing leadership, supportive policy environments and positive staff attitudes toward geriatric care were key enablers (Thillainadesan et al. 2021, 2022; Mistry et al. 2023; Ayoub et al. 2023). These factors created a climate in which new care models could be piloted and sustained. At the professional level, a major facilitator was geriatricians' belief in their specialised ability to manage complex needs, including acute complications, geriatric syndromes, delirium and functional decline. Their involvement was seen as critical for clinical outcomes, team confidence and care coordination (Thillainadesan et al. 2021). The team‐based nature of these models promoted interprofessional learning and capacity building. Geriatric engagement enhanced the competencies of other providers, embedding older adult care principles in surgical environments and improving patient safety and outcomes (Thillainadesan et al. 2021; Mistry et al. 2023).

We noted that in many studies, outcomes may not reflect what matters most to older surgical patients. While hospital length of stay was a standard metric—only a minority examined functional outcomes or quality of life. Few studies reported whether patients were discharged home. As the field evolves from quality improvement projects demonstrating shorter hospital stays to more rigorous systems implementation, we need evidence on how geriatric models of care improve outcomes that older adults value most. This review highlights the shared commitment to improving outcomes for older surgical patients through proactive, person‐centred multidisciplinary care. Future efforts should expand beyond orthopaedics, evaluate nurse‐led innovations and address systemic barriers.

## 5 Limitations

We acknowledge that restricting to English‐language publications may have excluded relevant studies, introducing language bias. Although the search was comprehensive, variability in terms describing older adults and models of care may have missed publications.

## 6 Conclusions

This review highlights the evolution of care models for older adults undergoing surgery and the significant yet underutilised role of nurses in improving outcomes. While orthopaedics has led the way in implementing such models, there is clear potential to extend these approaches to general, vascular, urology and colorectal surgery. Proactive, multidisciplinary, person‐centred care has been shown to improve patient outcomes, with advanced practice nurses playing a central role. Their leadership helps to bridge workforce gaps and enables more holistic care delivery—particularly in settings where geriatrician availability is limited. However, persistent barriers such as inadequate funding, staffing constraints and inconsistent adoption remain. Addressing these challenges will require coordinated efforts across policy, education and clinical practice. Future research should prioritise the evaluation of nurse‐led interventions and their impact on both patient outcomes and health system performance.

## Funding

The authors have nothing to report.

## Conflicts of Interest

The authors declare no conflicts of interest.

## Supporting information


**Data S1:** jan70518‐sup‐0001‐DataS1.pdf.

## Data Availability

The authors have nothing to report.
