# Supplementary material for: Geriatric Models of Surgical Care: A Scoping Review
Source: J Adv Nurs. 2026 Feb 2;82(9):8425–40. doi: 10.1111/jan.70518 (PMC13460995; doi:10.1111/jan.70518)

Supplementary Table 1: PRISMA-ScR checklist

SECTION	ITEM	PRISMA-ScR CHECKLIST ITEM	PAGE
**TITLE**			
Title	1	Identify the report as a scoping review.	0
**ABSTRACT**			
Structured summary	2	Provide a structured summary that includes (as applicable): background, objectives, eligibility criteria, sources of evidence, charting methods, results, and conclusions that relate to the review questions and objectives.	2
**INTRODUCTION**			
Rationale	3	Describe the rationale for the review in the context of what is already known. Explain why the review questions/objectives lend themselves to a scoping review approach.	5
Objectives	4	Provide an explicit statement of the questions and objectives being addressed with reference to their key elements (e.g., population or participants, concepts, and context) or other relevant key elements used to conceptualize the review questions and/or objectives.	6
**METHODS**			
Protocol and registration	5	Indicate whether a review protocol exists; state if and where it can be accessed (e.g., a Web address); and if available, provide registration information, including the registration number.	6
Eligibility criteria	6	Specify characteristics of the sources of evidence used as eligibility criteria (e.g., years considered, language, and publication status), and provide a rationale.	6-7
Information sources	7	Describe all information sources in the search (e.g., databases with dates of coverage and contact with authors to identify additional sources), as well as the date the most recent search was executed.	8
Search	8	Present the full electronic search strategy for at least 1 database, including any limits used, such that it could be repeated.	Supp Table 2
Selection of sources of evidence	9	State the process for selecting sources of evidence (i.e., screening and eligibility) included in the scoping review.	9
Data charting process	10	Describe methods of charting data from included sources of evidence (e.g., calibrated forms or forms been tested by the team before their use, and whether data charting was done independently or in duplicate) any processes for obtaining and confirming data from investigators.	9
Data items	11	List and define all variables for which data were sought and any assumptions and simplifications made.	9
Critical appraisal of individual sources of evidence	12	If done, provide a rationale for conducting a critical appraisal of included sources of evidence; describe the methods used and how this information was used in any data synthesis (if appropriate).	n/a
Synthesis of results	13	Describe the methods of handling and summarizing the data that were charted.	9
**RESULTS**			
Selection of sources of evidence	14	Give numbers of sources of evidence screened, assessed for eligibility, and included in the review, with reasons for exclusions at each stage, ideally using a flow diagram.	9-10

Characteristics of sources of evidence	15	For each source of evidence, present characteristics for which data were charted and provide the citations.	10
Critical appraisal within sources of evidence	16	If done, present data on critical appraisal of included sources of evidence (see item 12).	n/a
Results of individual sources of evidence	17	For each included source of evidence, present the relevant data that were charted that relate to the review questions and objectives.	10
Synthesis of results	18	Summarize and/or present the charting results as they relate to the review questions and objectives.	11-16
**DISCUSSION**			
Summary of evidence	19	Summarize the main results (including an overview of concepts, themes, and types of evidence available), link to the review questions and objectives, and consider the relevance to key groups.	16-19
Limitations	20	Discuss the limitations of the scoping review process.	19
Conclusions	21	Provide a general interpretation of the results with respect to the review questions and objectives, as well as potential implications and/or next steps.	19-20
**FUNDING**			

Supplementary Table 2: Complete search strategy

CINHAL	Search terms
Limiter	Limiters - Publication Date: 20150101-; English Language; Peer Reviewed; Human
Expanders	Expanders - Apply equivalent subjects, Search modes - Proximity
S37	S33 AND S34 AND S35 AND S36
S36	S1 OR S2 OR S3 OR S4 OR S5 OR S6 OR S7
S35	S22 OR S23 OR S24 OR S25 OR S26 OR S27 OR S28 OR S29
S34	S8 OR S9 OR S10 OR S11 OR S12 OR S13 OR S14 OR S15 OR S16 OR S17 OR S18 OR S19 OR S20 OR S21
S33	S30 OR S31 OR S32
S32	TI inpatient OR AB inpatient
S31	TI "acute care" OR AB "acute care"
S30	TI hospital* OR AB hospital*
S29	TI post#operativ* OR AB post#operativ*
S28	TI pre#operativ* OR AB pre#operativ*
S27	TI peri#operativ* OR AB peri#operativ*
S26	TI operat* OR AB operat*
S25	TI surgical OR AB surgical
S24	TI surger* OR AB surger*
S23	(MH "Perioperative Care+")
S22	(MH "Surgery, Operative+")
S21	TI "geriatric assessment" OR AB "geriatric assessment"
S20	TI "gerontololgic care model*" OR AB "gerontologic care model*"
S19	TI “gerontologic model* of care” OR AB “gerontologic model* of care”
S18	TI “geriatric care model*” OR AB “geriatric care model*”
S17	TI “geriatric model* of care” OR AB “geriatric model* of care”
S16	TI “multidisciplinary team*” OR AB “multidisciplinary team*”
S15	TI “multidisciplinary care” OR AB “multidisciplinary care”
S14	TI “care delivery model*” OR AB “care delivery model*”
S13	TI “integrated care” OR AB “integrated care”

S12	TI “integrated model*” OR AB “integrated model*”
S11	TI “care model*” OR AB “care model*”
S10	TI “model* of care” OR AB “model* of care”
S9	(MH "Gerontologic Care")
S8	(MH "Health Care Delivery, Integrated") OR (MH "Health Care Delivery+")
S7	TI "advanced age" OR AB "advanced age"
S6	TI gerontolog* OR AB gerontolog*
S5	TI "older patient*" OR AB "older patient*"
S4	TI "older adult*" OR AB "older adult*"
S3	TI elderly OR AB elderly
S2	TI geriatric* OR AB geriatric*
S1	(MH "Geriatrics+")
**Medline**	**Search terms**
Limiter	Peer Reviewed; Publication Date: 20150101-; English Language; Human
Expanders	Apply equivalent subjects, Search modes - Proximity
S36	S32 AND S33 AND S34 AND S35
S35	S8 OR S9 OR S10 OR S11 OR S12 OR S13 OR S14 OR S15 OR S16 OR S17 OR S18 OR S19 OR S20
S34	S1 OR S2 OR S3 OR S4 OR S5 OR S6 OR S7
S33	S21 OR S22 OR S23 OR S24 OR S25 OR S26 OR S27 OR S28
S32	S29 OR S30 OR S31
S31	TI inpatient OR AB inpatient
S30	TI "acute care" OR AB "acute care" S29 TI hospital* OR AB hospital*
S28	TI post#operativ* OR AB post#operativ*
S27	TI pre#operativ* OR AB pre#operativ*
S26	TI peri#operativ* OR AB peri#operativ*
S25	TI operat* OR AB operat*
S24	TI surgical* OR AB surgical*
S23	TI surger* OR AB surger*
S22	(MH "Perioperative Care+")
S21	(MH "General Surgery")

S20	TI “geriatric assessment” OR AB “geriatric assessment”
S19	TI “gerontololgic model* of care” OR AB “gerontologic model* of care”
S18	TI “gerontologic care model*” OR AB “gerontologic care model*”
S17	TI “geriatric care model*” OR AB “geriatric care model*”
S16	TI “geriatric model* of care” OR AB “geriatric model* of care”
S15	TI “multidisciplinary team*” OR AB “multidisciplinary team*”
S14	TI “multidisciplinary care” OR AB “multidisciplinary care”
S13	TI “care delivery model*” OR AB “care delivery model*”
S12	TI “integrated care” OR AB “integrated care”
S11	TI “integrated model” OR AB “integrated model”
S10	TI “care model*” OR AB “care model*”
S9	TI “model* of care” OR AB “model* of care”
S8	(MH "Delivery of Health Care, Integrated+") OR (MH "Delivery of Health Care+")
S7	TI “advanced age” OR AB “advanced age”
S6	TI gerontolog* OR AB gerontolog*
S5	TI "older patient*" OR AB "older patient*"
S4	TI "older adult*" OR AB "older adult*"
S3	TI elderly OR AB elderly
S2	TI geriatric* OR AB geriatric* S1 (MH "Geriatrics+")
**AgeLine**	**Search terms**
S37	S33 AND S34 AND S35 AND S36
S36	S1 OR S2 OR S3 OR S4 OR S5 OR S6
S35	S30 OR S31 OR S32
S34	S22 OR S23 OR S24 OR S25 OR S26 OR S27 OR S28 OR S29
S33	S7 OR S8 OR S9 OR S10 OR S11 OR S12 OR S13 OR S14 OR S15 OR S16 OR S17 OR S18 OR S19 OR S20 OR S21
S32	TI inpatient OR AB inpatient
S31	TI "acute care" OR AB "acute care"
S30	TI hospital* OR AB hospital*
S29	TI post#operativ* OR AB post#operativ*

S28	TI pre#operativ* OR AB pre#operativ*
S27	TI peri#operativ* OR AB peri#operativ*
S26	TI "perioperative care" OR AB "perioperative care"
S25	TI operat* OR AB operat*
S24	TI surgical* OR AB surgical*
S23	TI surger* OR AB surger*
S22	TI "general surgery" OR AB "general surgery"
S21	TI "geriatric assessment" OR AB "geriatric assessment"
S20	TI "gerontologic model* of care" OR AB "gerontologic model* of care"
S19	TI "gerontologic care model*" OR AB "gerontologic care model*"
S18	TI "geriatric care model*" OR AB "geriatric care model*"
S17	TI "geriatric model* of care" OR AB "geriatric model* of care"
S16	TI "multidisciplinary care" OR AB "multidisciplinary care"
S15	TI "multidisciplinary team*" OR AB "multidisciplinary team*"
S14	TI "care delivery model*" OR AB "care delivery model*"
S13	TI "integrated care" OR AB "integrated care"
S12	TI "integrated model*" OR AB "integrated model*"
S11	TI "care model*" OR AB "care model*"
S10	TI "model* of care" OR AB "model* of care"
S9	TI "gerontologic care*" OR AB "gerontologic* care"
S8	TI "delivery of health care" OR AB "delivery of health care"
S7	TI "delivery of health care, integrated" OR AB "delivery of health care, integrated"
S6	TI "advanced age" OR AB "advanced age"
S5	TI gerontolog* OR AB gerontology
S4	TI "older patient*" OR AB "older patient*"
S3	TI "older adult*" OR AB "older adult*"
S2	TI elderly OR AB elderly
S1	TI geriatric* OR AB geriatric*
**Embase**	**Search terms**

#40	#34 AND #35 AND #36 AND #37 AND [humans]/lim AND [english]/lim AND [embase]/lim AND [2015-2025]/py
#39	#35 AND #36 AND #37
#38	#34 AND #35 AND #36 AND #37
#37	#20 OR #21 OR #22 OR #23 OR #24 OR #25 OR #26 OR #27 OR #28 OR #29 OR #30
#36	#7 OR #8 OR #9 OR #10 OR #11 OR #12 OR #13 OR #14 OR #15 OR #16 OR #17 OR #18 OR #19
#35	#1 OR #2 OR #3 OR #4 OR #5 OR #6
#34	#31 OR #32 OR #33
#33	inpatient:ti,ab
#32	'acute care':ti,ab
#31	hospital*:ti,ab
#30	'post-operativ*':ti,ab
#29	'postoperativ*':ti,ab
#28	'pre-operativ*':ti,ab
#27	'preoperativ*':ti,ab
#26	'peri-operativ*':ti,ab
#25	perioperativ*:ti,ab
#24	operat*:ti,ab
#23	surgical*:ti,ab
#22	surger*:ti,ab
#21	'perioperative care'/exp OR 'perioperative care':ti,ab
#20	'surgery'/exp OR 'surgery':ti,ab
#19	'geriatric assessment':ti,ab
#18	'gerontologic model* of care':ti,ab
#17	'gerontologic care model*':ti,ab
#16	'geriatric care model*':ti,ab
#15	'geriatric model* of care':ti,ab
#14	'multidisciplinary team*':ti,ab
#13	'multidisciplinary care':ti,ab
#12	'care delivery model*':ti,ab

#11	'integrated care':ti,ab
#10	'integrated model*':ti,ab
#9	'care model*':ti,ab
#8	'model* of care':ti,ab
#7	'geriatric care'/exp OR 'geriatric care':ti,ab
#6	'advanced age':ti,ab
#5	gerontolog*:ti,ab
#4	'older patient*':ti,ab
#3	'older adult*':ti,ab
#2	elderly:ti,ab
#1	'geriatrics'/exp OR geriatrics:ti,ab
**Scopus**	**Search terms**
S6	S1 AND S2 AND S# AND S4 AND S5
S5	PUBYEAR > 2014 AND PUBYEAR < 2026 AND (LIMIT-TO (DOCTYPE , "ar")) AND (LIMIT-TO (LANGUAGE , "English")) AND (LIMIT-TO (EXACTKEYWORD , "Human"))
S4	(ABS (geriatric OR elderly OR "older adult" OR "older patient" OR gerontolog* OR "advanced age")
S3	ABS ("model* of care" OR "care model*" OR "integrated model*" OR "integrated care" OR "care delivery model*" OR "multidisciplinary care" OR "multidisciplinary team*" OR "geriatric model* of care" OR "geriatric care model*" OR "gerontologic care model*" OR "gerontologic model* of care" OR "geriatric assessment")
S2	ABS (surger* OR surgical OR operat* OR peri*operativ* OR pre*operativ* OR post*operativ*)
S1	ABS (hospital* OR "acute care" OR inpatient))
**Cochrane Library**	**Search terms**
#1	MeSH descriptor: [Geriatrics] explode all trees
#2	(geriatric*):ti,ab,kw OR (elderly):ti,ab,kw OR (NEXT older adult*):ti,ab,kw OR (NEXT older patient*):ti,ab,kw (Word variations have been searched)
#3	(gerontolog*):ti,ab,kw OR ("advanced age"):ti,ab,kw (Word variations have been searched)
#4	MeSH descriptor: [Delivery of Health Care] explode all trees
#5	MeSH descriptor: [Delivery of Health Care, Integrated] explode all trees
#6	(NEXT model* of care):ti,ab,kw OR (NEXT care model*):ti,ab,kw OR ("integrated care"):ti,ab,kw OR (NEXT integrated model*):ti,ab,kw OR (NEXT care delivery model*):ti,ab,kw (Word variations have been searched)

#7	("multidisciplinary care"):ti,ab,kw OR ("multidisciplinary team"):ti,ab,kw OR (NEXT geriatric model* of care):ti,ab,kw OR (NEXT geriatric care model*):ti,ab,kw OR (NEXT gerontologic care model*):ti,ab,kw (Word variations have been searched)
#8	(NEXT gerontologic model* of care):ti,ab,kw OR ("geriatric assessment"):ti,ab,kw (Word variations have been searched)
#9	MeSH descriptor: [General Surgery] explode all trees
#10	MeSH descriptor: [Perioperative Care] explode all trees
#11	(surger*):ti,ab,kw OR (surgical*):ti,ab,kw OR (operat*):ti,ab,kw (Word variations have been searched)
#12	(perioperativ*):ti,ab,kw OR (peri-operativ*):ti,ab,kw (Word variations have been searched)
#13	(preoperativ*):ti,ab,kw OR (pre-operativ*):ti,ab,kw (Word variations have been searched)
#14	(postoperativ*):ti,ab,kw OR (post-operativ*):ti,ab,kw (Word variations have been searched)
#15	(hospital*):ti,ab,kw OR ("acute care"):ti,ab,kw OR (inpatient):ti,ab,kw (Word variations have been searched)
#16	#1 OR #2 OR #3
#17	#4 OR #5 OR #6 OR #7 OR #8
#18	#9 OR #10 OR #11 OR #12 OR #13 OR #14
#19	#16 AND #17 AND #18 AND #15 with Cochrane Library publication date Between Jan 2015 and Feb 2025

Supplementary Table 3. Geriatric models of surgical care: intervention components

Lead author, year	Geriatric ward /specific beds	Ward rounds	Multi-disciplinary team meetings	Hospital staff education geriatric care	Fast track ED protocol	Medical sub-speciality referrals	Pre-op assessment or optimisation	Clinical investigation/diagnostic tests	Shared goal setting, individual care plan	Advanced care planning	Surgical prioritisation/ access to surgery	Optimisation of			Prevention and/or Management of					Patient & family-centred education counselling & discharge planning	Early mobilisation	Geriatric rehab or prehabilitation	Care pathway	Follow-up care post discharge led by the geriatric team
												Comorbidities	Nutrition and fluid	Medication	Delirium	Urinary or bowel complications	Thrombosis and pressure injury	Pain	Anaemia					
Wallace et al., 2019		X	X		X	X							X			X	X	X	X	X	X		X	
Duaso et al., 2018	X	X			X		X					X		X	X		X	X		X	X		X	
Kregel et al., 2024	X		X				X					X		X	X		X	X					X	
Soong et al., 2016		X	X			X	X							X	X		X	X		X	X	X	X	
Wu et al., 2023			X				X	X			X			X			X	X		X	X	X	X	
Khadaroo et al., 2020	X													X	X	X				X	X		X	
Shigemoto et al., 2021					X								X	X	X	X					X	X	X	
Sim et al., 2023						X	X	X	X			X	X					X	X	X		X	X	X
Kusen et al., 2021	X	X									X								X	X			X	
Werner et al., 2020		X									X								X				X	
Lester et al., 2022			X			X	X		X				X	X	X			X		X	X	X	X	X
Chebl et al., 2024				X		X	X		X			X	X	X	X			X		X			X	X
Lamb et al., 2017	X					X						X	X					X		X	X		X	
Murphy et al., 2019					X		X											X					X	
Neuerburg et al., 2019	X	X	X												X						X	X	X	
Kusen et al., 2019		X				X		X			X	X	X	X	X						X	X	X	
Morris et al., 2023		X	X		X			X	X						X					X	X	X	X	
Asplin et al., 2017	X	X	X						X											X	X	X	X	
Nijmeijer et al., 2018	X	X	X		X			X	X		X		X	X						X			X	
Peng et al., 2020	X										X												X	
DeLas Casas et al., 2021		X	X				X					X								X			X	
Lau et al., 2017																							X	

												Optimisation of			Prevention and/or Management of									
**Lead author, year**	**Geriatric ward /specific beds**	**Ward rounds**	**Multi-disciplinary team meetings**	**Hospital staff education geriatric care**	**Fast track ED protocol**	**Medical sub-speciality referrals**	**Pre-op assessment or optimisation**	**Clinical investigation/diagnostic tests**	**Shared goal setting, individual care plan**	**Advanced care planning**	**Surgical prioritisation/ access to surgery**	**Comorbidities**	**Nutrition and fluid**	**Medication**	**Delirium**	**Urinary or bowel complications**	**Thrombosis and pressure injury**	**Pain**	**Anaemia**	**Patient & family-centred education counselling & discharge planning**	**Early mobilisation**	**Geriatric rehab or prehabilitation**	**Care pathway**	**Follow-up care post discharge led by the geriatric team**
Lee et al., 2021												X											X	
Pankratz et al., 2023			X				X		X			X	X							X		X	X	
Tarazona-Santabalbina et al., 2019		X										X	X	X	X	X	X	X	X	X				
Shahrokni et al., 2020		X				X	X					X		X	X	X	X	X		X	X			
Sura-Amonrattana et al., 2021							X				X	X	X		X	X	X	X		X	X			
Thillainadesan et al., 2022		X	X	X		X			X				X	X	X	X	X	X		X	X			
Solberg et al., 2023	X	X					X	X			X	X		X	X	X	X				X			
Zhou et al., 2024			X		X		X	X			X	X	X	X	X		X	X	X	X				
Mcdonald et al., 2018		X					X		X			X	X	X	X		X	X		X				
Ayoub et al., 2023							X					X		X			X	X		X				
Liu et al., 2022	X	X	X		X		X	X				X	X				X			X	X			
Thu et al., 2021	X	X	X			X	X					X	X	X	X	X		X		X	X	X		
Natesan et al., 2022		X				X	X		X			X	X	X		X		X		X	X	X		
Van Der Vlies et al., 2020			X			X	X							X	X				X	X		X		
Ramírez-Martín et al., 2022		X	X				X	X	X			X	X	X	X				X			X		X
Meulenbroek et al., 2024							X						X		X				X			X		
Lin et al., 2021		X		X			X				X			X	X			X		X				X
Liu et al., 2024	X						X	X				X	X					X				X		
Cizginer et al., 2023		X				X		X	X			X		X				X		X	X			
Giannotti et al., 2022		X					X					X	X	X				X		X	X			
Thingstad et al., 2016	X						X		X			X			X					X	X	X		
Kusen et al., 2022	X	X			X		X	X			X	X		X	X					X	X			

Lead author, year	Geriatric ward /specific beds	Ward rounds	Multi-disciplinary team meetings	Hospital staff education geriatric care	Fast track ED protocol	Medical sub-speciality referrals	Pre-op assessment or optimisation	Clinical investigation/diagnostic tests	Shared goal setting, individual care plan	Advanced care planning	Surgical prioritisation/ access to surgery	Optimisation of			Prevention and/or Management of					Patient & family-centred education counselling & discharge planning	Early mobilisation	Geriatric rehab or prehabilitation	Care pathway	Follow-up care post discharge led by the geriatric team
												Comorbidities	Nutrition and fluid	Medication	Delirium	Urinary or bowel complications	Thrombosis and pressure injury	Pain	Anaemia					
Neupane et al., 2023		X	X	X								X		X	X									
Checa-López et al., 2024		X					X		X			X	X	X	X					X		X		
Festen et al., 2020			X						X				X		X							X		
Breda et al., 2023						X		X	X		X			X	X					X		X		X
De Bueck et al., 2024							X					X			X						X	X		
Gilbert et al., 2021	X			X			X	X					X											
Middleton et al., 2016	X	X	X				X				X	X										X		X
Pablos-Hernández et al., 2020	X																							
Lisk et al., 2023	X	X			X						X											X		
Xu et al., 2024	X				X		X	X				X		X							X	X		X
Coskun & Duygulu, 2021							X					X	X							X		X		X
Hofmeister et al., 2020																								
Hu et al., 2022		X												X						X				
Shipway et al., 2018		X	X																	X		X		
Irimia et al., 2022						X		X		X		X		X						X				
Thillainadesan et al., 2021																								
Fagard et al., 2023																								
Adogwa et al., 2018							X					X										X		
Owen et al., 2023																								
Lynch et al., 2015		X	X				X													X				
Noticewala et al., 2016		X					X					X		X										
Forni et al., 2016																				X				
Henderson et al., 2017		X										X												

Lead author, year	Geriatric ward /specific beds	Ward rounds	Multi-disciplinary team meetings	Hospital staff education geriatric care	Fast track ED protocol	Medical sub-speciality referrals	Pre-op assessment or optimisation	Clinical investigation/diagnostic tests	Shared goal setting, individual care plan	Advanced care planning	Surgical prioritisation/ access to surgery	Optimisation of			Prevention and/or Management of					Patient & family-centred education counselling & discharge planning	Early mobilisation	Geriatric rehab or prehabilitation	Care pathway	Follow-up care post discharge led by the geriatric team
												Comorbidities	Nutrition and fluid	Medication	Delirium	Urinary or bowel complications	Thrombosis and pressure injury	Pain	Anaemia					
Tseng et al., 2019						X														X		X		X
Baroni et al., 2019		X	X			X	X													X		X		
Tseng et al., 2021				X			X													X		X		
Van Leendert et al., 2021		X		X			X					X	X	X						X		X		
Ogawa et al., 2022		X										X												
Higashikawa et al., 2022														X										
Liang et al., 2023			X				X	X			X	X									X	X		
Mistry et al., 2023																								
Bremen et al., 2024																								
Leede et al., 2020		X					X					X		X						X				
Braude et al., 2017		X	X	X																				
Partridge et al., 2017							X							X										
Partridge et al., 2021																				X		X		
Wang et al., 2023		X	X	X										X						X				

Supplementary Figure 1. Countries included in published studies

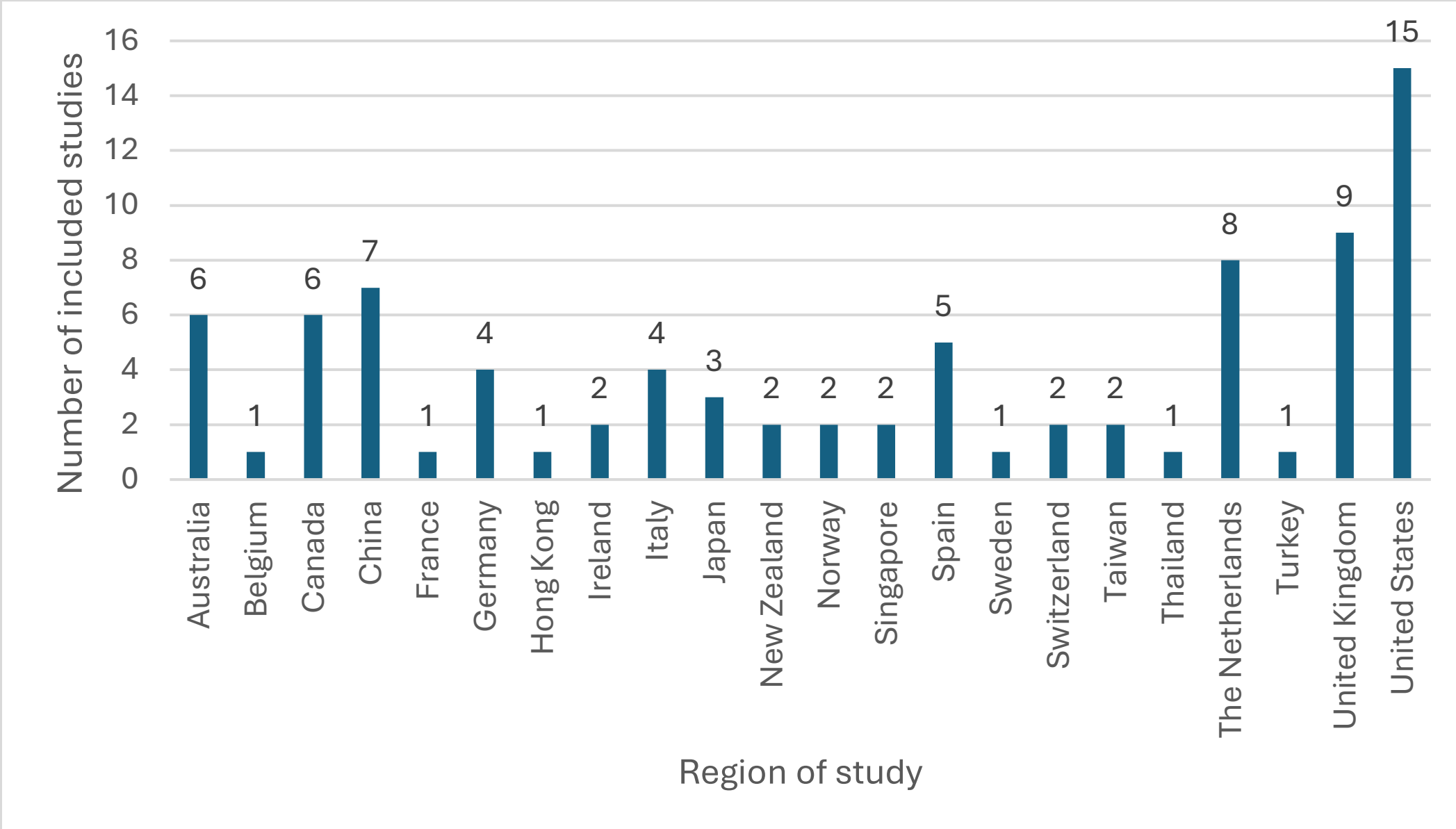

Supplementary Figure 2. Commencement point of model of care by surgical speciality

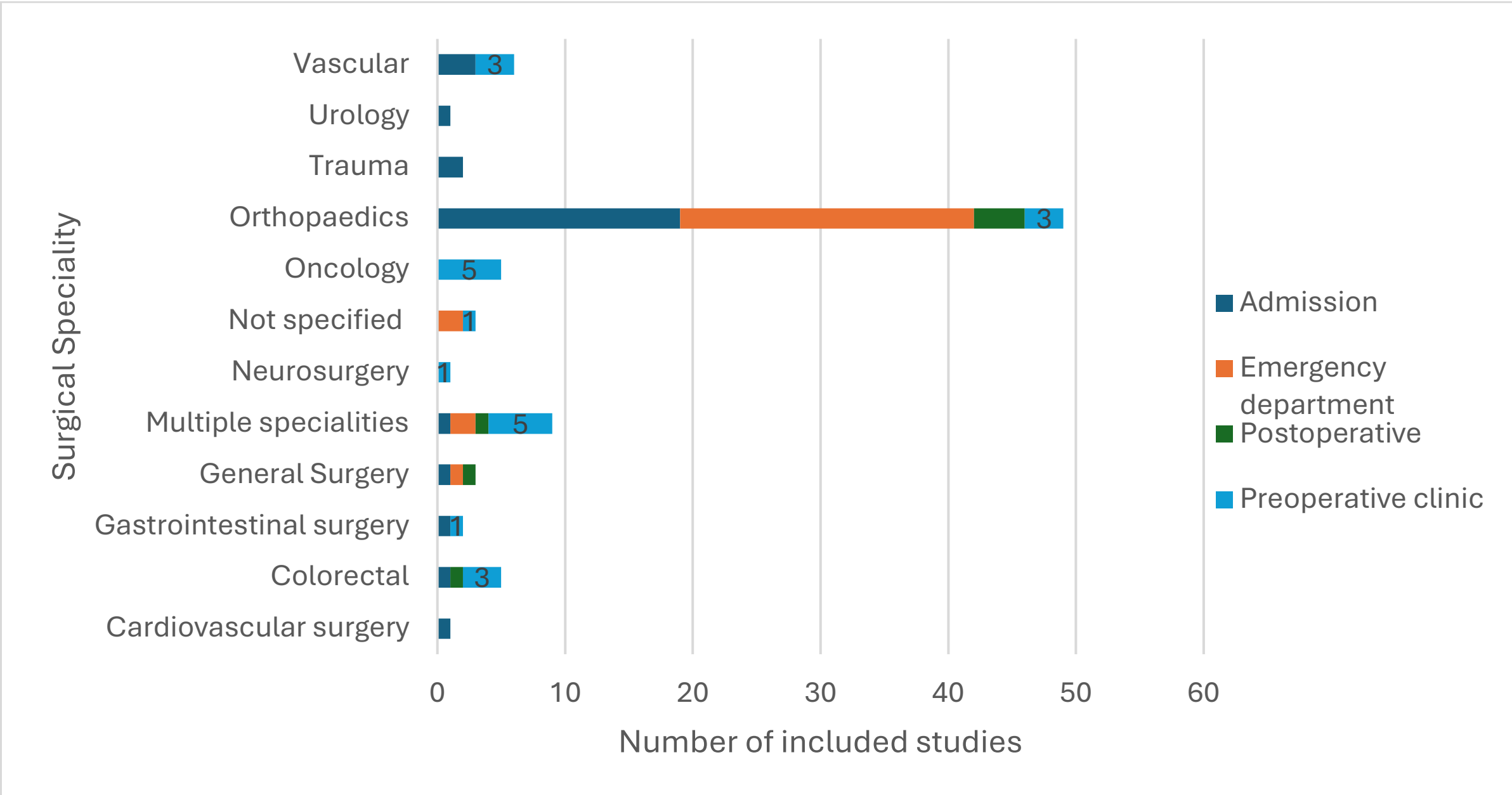

Supplementary Figure 3. Commencement point of model of care location: (A) including orthopaedic studies" (B) excluding orthopaedic studies

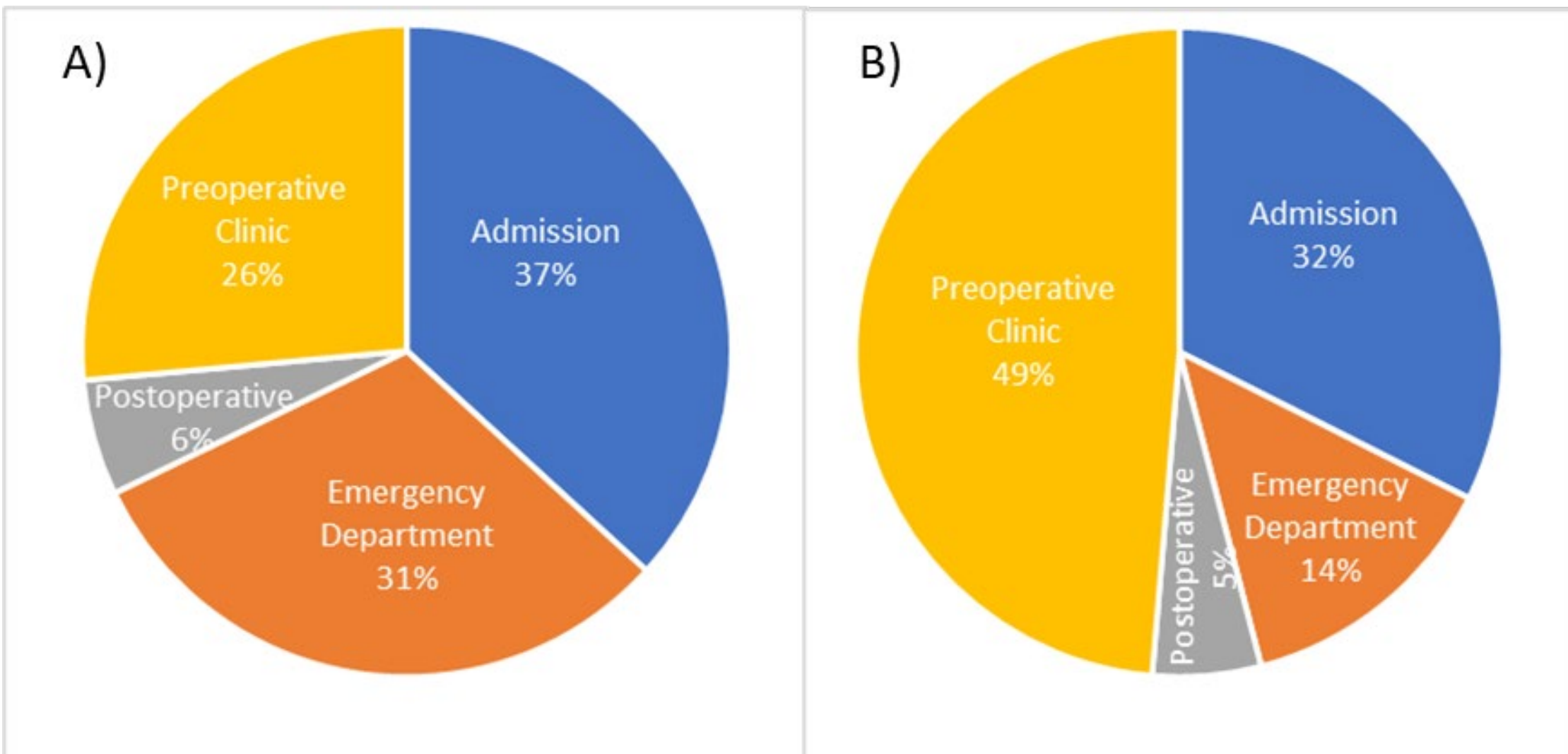

Supplement: Supplementary file 1 [file JAN-82-8425-s001.pdf]
